# Unraveling the novel effects of aroma from small molecules in preventing hen egg white lysozyme amyloid fibril formation

**DOI:** 10.1371/journal.pone.0189754

**Published:** 2018-01-22

**Authors:** Zahra Seraj, Arefeh Seyedarabi, Ali Akbar Saboury, Mehran Habibi-Rezaei, Shahin Ahmadian, Atiyeh Ghasemi

**Affiliations:** 1 Department of Biochemistry, Institute of Biochemistry and Biophysics, University of Tehran, Tehran, Iran; 2 School of Biology, University College of Science, University of Tehran, Tehran, Iran; INRA Centre de Jouy-en-Josas, FRANCE

## Abstract

This study investigated for the first time the molecular effectiveness of 'aroma' from three small molecules including a phenol (phenyl ethyl alcohol; PEA) and an aldehyde (cinnamaldehyde; Cin) both containing an aromatic ring, and a diamine (N,N,N,N'- Tetramethylethylenediamine; TEMED) at two different amounts (small; S and large; L) in preventing hen egg white lysozyme (HEWL) amyloid fibril formation using Thioflavin T and Nile red fluorescence assays, circular dichroism spectroscopy, SDS-polyacrylamide gel electrophoresis, atomic force microscopy, dynamic light scattering and HEWL activity test. Interestingly, the results revealed that (1) the aroma of PEA, identified as an active constituent of *Rosa damascena*, prevented fibril formation since PEA-L was able to trap the oligomeric form of HEWL in contrast to PEA-S where protofibrils but not mature fibrils were formed; (2) Cin, previously shown to prevent fibril formation in the liquid form, was also shown to do so in the aroma form by producing protofibrils and not mature fibrils in both Cin- L and Cin-S aroma forms and (3) the aroma of TEMED-L was able to retain HEWL’s native structure completely and prevented both aggregation and fibril formation, while TEMED-S prevented HEWL fibril formation and instead directed the pathway towards amorphous aggregate formation. Furthermore, the ability to trap oligomeric species (by PEA-L aroma) is of great importance for further research as it provides routes for preventing the formation of toxic oligomeric intermediates along the fibrillation pathway. Last but not least, the novelty of this *in vitro* study on the effect of aroma at the molecular level with a unique experimental set-up using HEWL as a model protein in assessing amyloid fibril formation paves the way for more and detailed studies on the importance of aroma producing molecules and their effects.

## Introduction

**Fig 1:**
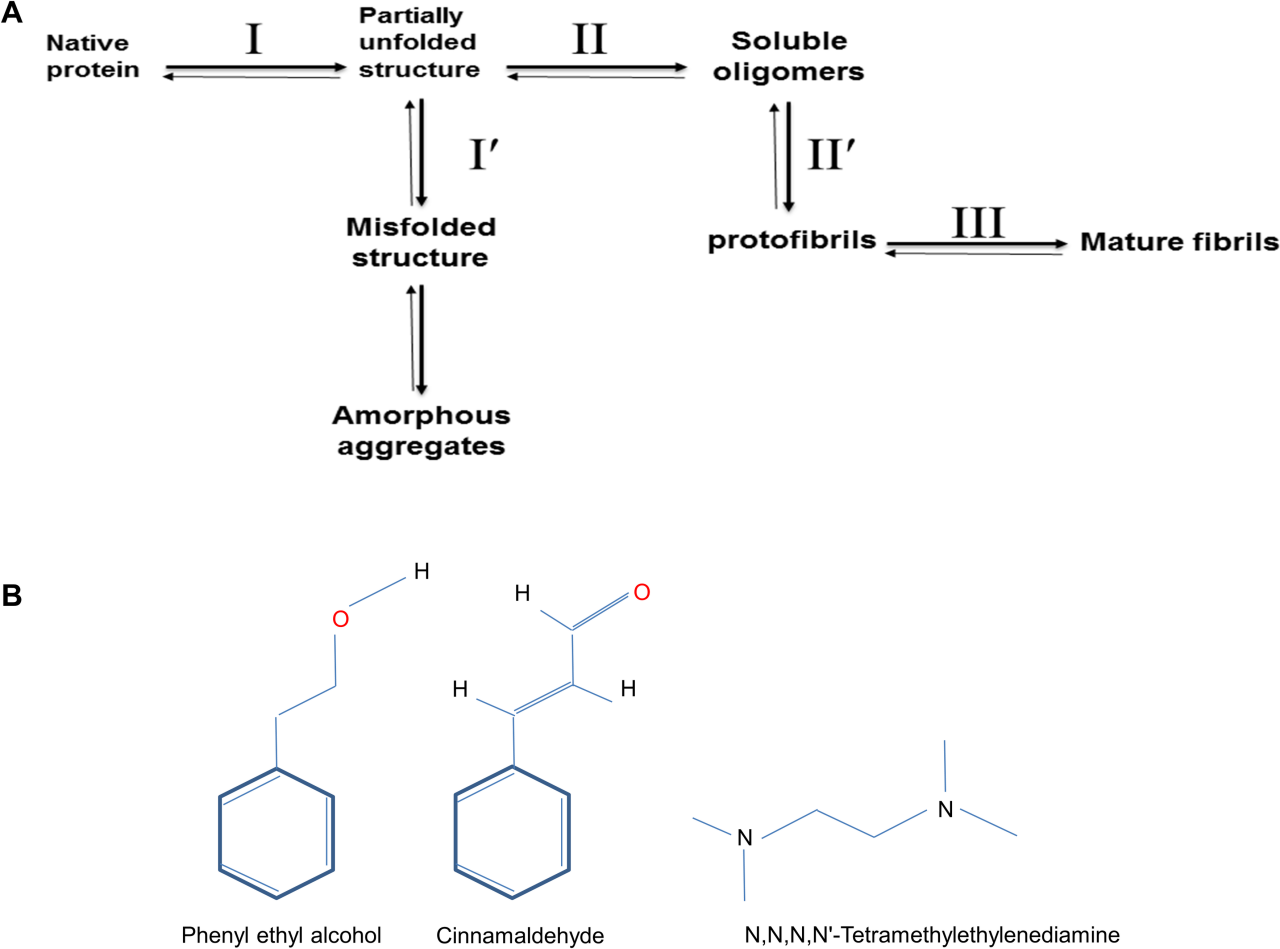
A preview of the protein aggregation/fibrillation pathway and chemical structures of the small aroma producing molecules used in this study. (A) A general overview of the steps involved in globular protein aggregation/fibrillation. The pathway of assembly varies in different references, details may change i.e. some references use protofibrils as synonym for oligomers [9], however the theory generally holds to be reliable. (B) 2D chemical structures of the two polyphenols and the polyamine used in this study.

A great body of evidence has suggested that there is a strong connection between amyloid fibril formation and disease pathology [1]. Amyloid fibrils are large and ordered aggregates [2], containing a universal “cross-β” core structure composed of arrays of β-sheets running parallel to the long axis of the fibrils [3]. Until now, about 20 different diseases have been described which are associated with changes in the solubility of a protein or protein fragment from native soluble, into insoluble aggregates or plaques which deposit in a variety of organs and tissues causing amyloidosis [4]. Amyloeidogenic proteins can be categorised as two types; 1- globular proteins with known tertiary structures and 2- proteins without known tertiary structures (intrinsically unfolded). Proteins with a native globular structure become partially unfolded (Fig 1A, step II) when exposed to harsh conditions *in vitro* such as high temperature and acidic pH [5], leading to the formation of amorphous aggregates (Fig 1A, step I′), a kind of aggregation without any specific structures, or to the formation of oligomers with increased beta conformations. Oligomeric (globular) intermediates may assemble to form soluble protofibrillar structures [5] with 2–5 nm diameter or an approximate width size of 2.5–3.5 nm (Fig 1A, step II) [6–8]. In the final step, if protofibrils are associated laterally or twisted together they can make insoluble mature fibrils (Fig 1A, step III) known as amyloid fibrils with a diameter size of 4–13 nm or an approximate width size of 7.5–8.0 nm [6–8]. Therefore, it is understood that blocking any of these steps or directing the pathways towards steps I′ or II′ (Fig 1A) would hinder formation of amyloid fibrils.

On this basis, several therapeutic approaches have been suggested for the treatment of amyloidogenic diseases including (a) reduction in the production of the amyloidogenic form of proteins (by blocking step III); (b) increasing the clearance rate of misfolded or aggregated proteins; (c) increasing the stability of the native state of amyloidogenic proteins (by blocking step I); and (d) directly inhibiting the self-assembly process [10]. Amongst these approaches, great effort has been directed in the recent years towards screening or synthesising compounds that interfere with fibrillation to overcome amyloidosis [11–13]. Numerous compounds such as proteins, peptides and small molecules have therefore been studied and reported to have an ability to prevent amyloidogenic protein fibrillation, by binding to amyloid fibrils and thereby blocking further polymerisation [14,15]. Additionally, it has been suggested that small molecules composed of aromatic rings such as polyphenols play an effective role in inhibiting amyloid formation [9,16,17]. Polyphenols are a large group of natural and synthetic small molecules that are composed of one or more aromatic phenolic rings [13]. Given the fact that aromatic residues are generally present in proteins forming amyloids, it has been suggested that the interference of aromatic stacking or ח-ח interactions by such polyphenolic compounds may play a major role in preventing amyloid fibril formation [1,13,18,19]. Hence, polyphenols have been suggested as good inhibitors in preventing fibril formation in several studies [16,20–22]. On the other hand, there are reports of another group of small organic molecules, polyamines, which prevent protein aggregation [23,24]. Polyamines are multivalent cations including aliphatic hydrocarbon chains separating the charges, and thus could potentially bind to proteins via both hydrophobic and electrostatic interactions [25].

The use of aroma in diseases is being practiced in aromatherapy, which is a subdivision of phytotherapy making use of natural pure essential oils from aromatic plants (such as peppermint, rose, lavender and rosemary) to help relieve health problems and improve the quality of life in general [26]. Aromatherapy has been used to promote relief of pain, reduce disturbed behaviour, promote relaxation and sleep, reduce depressive symptoms and stimulate motivational behaviours [26]. Although there are some evidences showing the effect of aroma in controlling central nervous system [27–29], the therapeutic effect of aroma and aromatherapy are not at all supported by molecular studies [30].

In this study, three different compounds (a phenol, an aldehyde and a diamine) were investigated for their effectiveness of aroma in preventing hen egg white lysozyme (HEWL) amyloid fibril formation in two different amounts (S; small and L; large). The phenol used was phenyl ethyl alcohol (PEA) from *Rosa Damascena* [31], which gives the rose flower its pleasant smell. The aldehyde was cinnamaldehyde (Cin) from *Cinnamomum zelanicum*, which is an aldehyde giving cinnamon its flavour as well as odour [32]. The complete *Rosa Damascena* rose flower extract (including petals and leaflets) and cinnamaldehyde both in solution have been shown in previous studies to inhibit fibril formation [33–35]. The diamine used was N,N,N,N’-Tetramethylethylenediamine (TEMED), which is a water-white coloured liquid with a fishlike odour [36]. TEMED was chosen as a readily available on the shelf chemical (mainly used in SDS-PAGE for setting polyacrylamide gels) having the required properties as a polyamine with an odour for comparison with the two other compounds. The chemical structures of the three compounds used in this study are shown in Fig 1B. PEA and Cin both have a single aromatic ring while TEMED is a linear non-aromatic structure containing two amine groups. HEWL was used here as a model protein, which has the ability to self-assemble and has been used extensively to study protein stability, folding and aggregation [37].

Therefore, in this study the effect of ‘aroma’ of the three different compounds were assessed on HEWL amyloid fibril formation *in vitro* to understand the mechanism(s) involved at the molecular level. HEWL amyloid formation was assessed using atomic force microscopy (AFM), Thioflavin T (ThT) fluorescence assay and dynamic light scattering (DLS). The secondary structure and hydrophobic patch analyses were performed by circular dichroism (CD) and Nile red fluorescence assay, respectively. The relative enzymatic activity of HEWL was also assessed to further our understanding of the effect of aroma on inhibition of amyloid formation.

## Materials and methods

### Materials

Hen egg-white lysozyme (HEWL; catalogue number L6876), phenyl ethyl alcohol (PEA; catalogue number W285803), Thioflavin T (ThT), Nile red, *Micrococcus lysodeikticus* (ATCC No. 4698), glycine (CAS No. 56-40-6), potassium phosphate (catalog no. 90.22,130–9), sodium dodecyl sulphate (SDS; catalogue number 85,192–2), N,N,N,N'-Tetramethylethylenediamine (TEMED; CAS number 110–189) and Trans-cinnamaldehyde (Cin; Lot number MKBV8774V) were all purchased from Sigma-Aldrich. Excelband all blue regular range protein marker (PM1500) was purchased from SMOBIO. Mica for atomic force microscopy (catalogue number 92680) was purchased from PELCO. Acrylamide (UN-NO 2074) and N, N’methylendiacrylamid (EC number 203-750-9) were purchased from Merck.

### UV absorbance spectroscopy

UV–vis absorbance spectra were recorded using a UV-vis Spectrophotometer (Varian, Carry 100 Bio, Australia). The protein concentration was calculated using the extinction coefficient ε = 2.65 dm^3^g^−1^cm^−1^at λ = 280 nm [38]. Three scans were averaged for each sample.

### HEWL sample solution preparation and incubation studies

**Fig 2:**
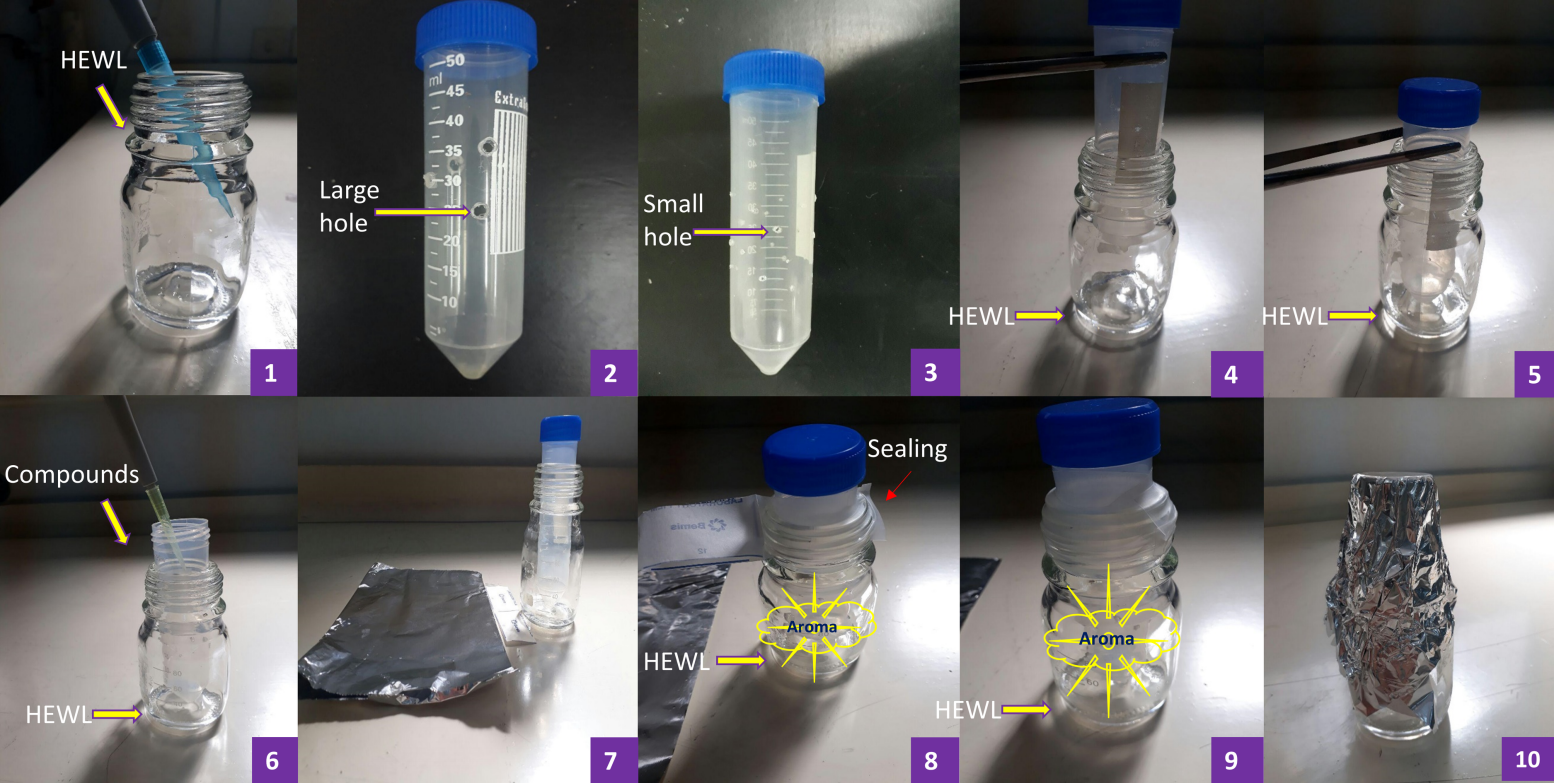
The experimental setup. (1) 5 ml of HEWL at 2 mg/ml was initially added to the bottom of a 100 ml Duran bottle; (2 and 3) Small and large holes were made in 50 ml falcon tubes, respectively; (4 and 5) The falcon tubes with holes were placed into Duran bottles containing HEWL; (6) 50 μl volume of PEA, Cin or TEMED were added to the falcon tubes with small (S) and large (L) holes and placed inside the Duran bottle containing HEWL; (7–10) The lids of the falcon tubes and Duran bottles where sealed together and incubated at 54°C for 24 hours in a shaker at 150 rpm for the process of aggregation or fibril formation to take place and the effect of the aroma of PEA, Cin or TEMED in preventing fibrillation to be assessed.

Sample solutions of HEWL at 2 mg/ml (concentration determined by UV spectrophotometer at a wavelength of 280 nm) in 50 mM glycine-HCl (pH 2.2) was used in this study. PEA, Cin and TEMED were all used in their original purchased forms. The experimental set-up (Fig 2) was such that 5 ml of 2 mg/ml HEWL was initially added to the bottom of an empty 100 ml Duran bottle; then 50 μl volume of aroma producing PEA, Cin or TEMED were added to empty 50 ml falcon tubes with small (S, average 0.15 mm diameter size) and large (L, average 0.45 mm diameter size) holes and placed inside the Duran bottle containing HEWL; the lids of the falcon tube and Duran bottle where sealed together. The Duran bottles containing HEWL as well as falcon tubes (having either S or L sized holes) with aroma producing compounds inside, were incubated at 54°C for 24 hours in a shaker at 150 rpm for the process of aggregation or fibril formation to take place. The S and L sized holes in falcon tubes represented the small or large amount of aroma diffused out in the Duran bottle where HEWL existed. It was noticeable that upon completion of the experiments a very small volume of compounds were reduced (2–5 μl), although a great amount of odour was spread in the Duran bottle environments outside of the falcon tubes containing HEWL. The positive control HEWL solution, referred to as ‘non-heated’ in this study, was also dissolved in 50 mM glycine-HCl (pH 2.2) at 2 mg/ml but was not incubated. Another control HEWL solution (a negative control), referred to as ‘non-treated’ also contained 2 mg/ml HEWL which was incubated for 24 hours but in the absence of any of the aroma producing compounds mentioned in this study. All the following experiments including ThT, Nile red, CD, SDS-PAGE, AFM, DLS and HEWL activity test were carried out using samples prepared and incubated from this stage.

### Thioflavin T (ThT) fluorescence assay

HEWL samples incubated with or without aroma were diluted 50-fold with ThT solution (at 25 μM) and fluorescence intensities were recorded at 484 nm after excitation at 440 nm. Excitation and emission slit widths were both set at 5 nm. Samples of not-heated and not-treated HEWL were also used and ThT recorded as positive and negative controls, respectively. The results were repeatable and standard deviation bars were calculated for the graph using multiple data.

### Nile red fluorescence assay

Nile red powder was initially dissolved in ethanol and then 5 μl of the Nile red solution (at 2 mM) was diluted in 990 μl acidic deionized water (pH = 1.6). 5 μl of HEWL sample post-incubation were added to the Nile red solution and incubated in a dark room for 20 min. The excitation wavelength was 553 nm and emission spectra were recorded between 600 and 700 nm. Excitation and emission slit widths were both set at 10 nm. The control samples were also recorded.

### Circular dichroism spectroscopy

Circular dichroism (CD) spectra of HEWL samples were recorded from 250 to 195 nm with an AVIV 215 spectrophotometer (Aviv Associates, Lakewood, NJ, USA). The HEWL samples post incubation were diluted 10 times for CD. CD spectrum of glycine buffer (pH 2.2) was recorded and subtracted from the CD spectra of HEWL samples post-incubation. Three scans of each sample was measured and averaged. The control buffer scans were run in duplicates, averaged, and then subtracted from the sample spectra. The results were plotted as ellipticity (deg. cm^2^ dmol^−1^) versus wavelength (nm). The control samples were also measured using CD.

### Protein gel electrophoresis

Tris-glycine SDS polyacrylamide gel electrophoresis (SDS-PAGE under reducing conditions to analyse the HEWL samples in this study. A pre-stained protein marker was used and 5 μl of each sample mixed with an equal volume of 2X loading buffer were loaded into an 18% gel.

### Atomic force microscopy (AFM)

AFM scans were performed using a Veeco AFM instrument (Sharif University, Tehran, Iran). For preparation of AFM samples, 10 μl of each sample was loaded onto a mica surface and allowed to fix on mica by incubation for 30 minutes at room temperature. The mica surface was then washed once with 100 μl deionized water and left to dry at room temperature before being examined by the atomic force microscope [39].

### Dynamic light scattering (DLS)

Dynamic light scattering measurements were performed using the Malvern Zeta sizer Nano ZS ZS (School of Pharmacy, Tehran University of Medical Sciences, Tehran, Iran). The apparatus was equipped with a laser with a wavelength of 633 nm. Samples were placed into disposable cuvettes with a 1 cm optical path. Scatter radiation was collected at an angle of 173° (backscatter mode). Each scan consisted of 70 runs and each run duration was 60 seconds. The temperature of the measurements was set at 25°C. The refractive index of the material (protein) and the absorption were set to 1.59 and 0.01, respectively. The following parameters were used: the dispersant viscosity was 0.8872 cP and the refractive index was 1.330.

### Lytic activity of HEWL

The rate of lysis of *Micrococcus lysodeikticus (M*. *luteus)* by HEWL was measured as reported [40]. The lytic activity was monitored turbidometrically at 450 nm at pH 6.2 and 25°C. To a 1 ml suspension of *M*. *luteus* in 0.1 mM of potassium phosphate buffer, 10 μl of the HEWL samples were added to a final concentration of 0.2 mg/ml. Changes in the turbidity at 450 nm was recorded per minute using a spectrophotometer. The two formulae [40] (see Eqs 1 and 2), which were used to obtain the activity are given below:

$$\mathrm{Unit}/\mathrm{ml}\;\mathrm{enzyme}=\frac{\left(\Delta A450/\min\;\mathrm{Test}–\Delta A450/\min\;\mathrm{Blank}\right)\;\left(\mathrm{df}\right)}{\left(0.001\right)x\left(0.01\right)}$$ (1)

df = dilution factor
0.001 = ΔA_450_ as per the Unit Definition
0.01 = Volume (in milliliters) of Enzyme Solution

$$\mathrm{Relative}\;\mathrm{activity}\%=\frac{\left(A450/\min0\;\mathrm{sample}–A450/\min5\;\mathrm{sample}\right)\;\left(100\right)}{\left(A450/\min0\;\mathrm{Not}–\mathrm{heated}–A450/\min5\;\mathrm{Not}–\mathrm{heated}\right)}$$ (2)

## Results

### Inhibition of HEWL fibril formation

**Fig 3:**
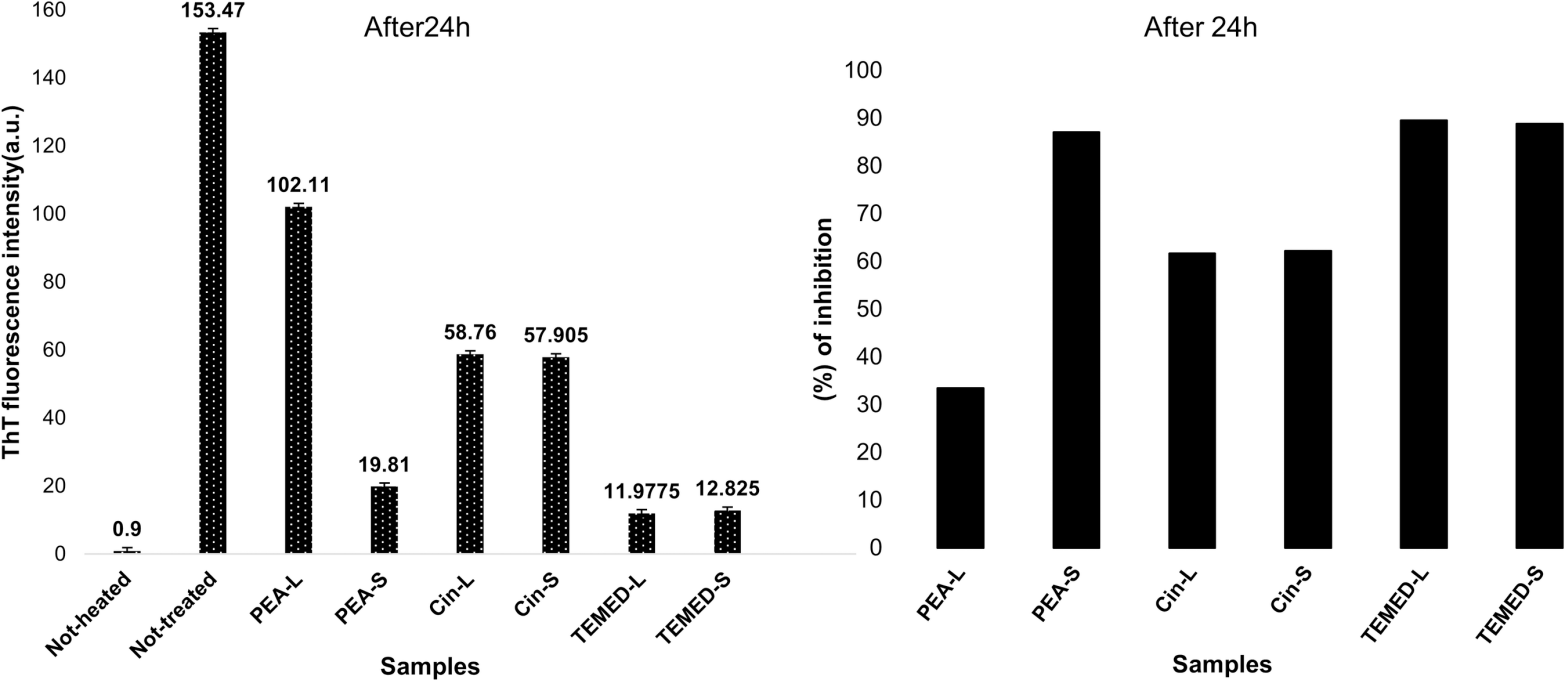
Inhibition of HEWL fibril formation. (A) Effect of PEA, Cin and TEMED aroma on HEWL amyloid fibrillation detected by ThT emission. (B) Percentage of inhibition in the presence of either small or large amounts of aroma on HEWL fibril formation. HEWL samples were incubated for 24 hours at pH 2.2 and 54°C (except for the not-heated sample) and ThT emission measured at 484 nm.

Three compounds were tested for their ability to inhibit amyloid fibril formation of HEWL by ThT fluorescence assay. It is known that ThT interacts rapidly and specifically with amyloid fibrils [41], and an increase in the ThT fluorescence signal is an important indicator of the presence of amyloid fibrils [41]. Therefore, to determine the anti-amyloidogenic efficiency of the different aroma against HEWL amyloid fibrillation, the amount of amyloid formation was measured in the presence and in the absence of PEA, Cin and TEMED aroma using the ThT binding assay. As it is shown in Fig 3A, PEA, Cin and TEMED were all able to decrease fibril formation. Whereas PEA-S, Cin-L and Cin-S were able to reduce fibrillation to some noticeable extent, TEMED showed remarkable and eye catching effects. The percentage of inhibition in the presence of different aroma was calculated based on the ThT emission intensity in the presence and absence of aroma (Fig 3B). It is clear that TEMED was able to inhibit fibril formation up to almost 90% and PEA-S, Cin-L and Cin-S inhibited fibril formation by more than 30%. PEA-L, on the other hand, showed the least reduction in ThT emission compared to the not-treated HEWL sample (minimal data set for ThT fluorescence assay results is available in S1 Fig).

### Analysis of hydrophobic patches and secondary structure of HEWL treated with aroma

**Fig 4:**
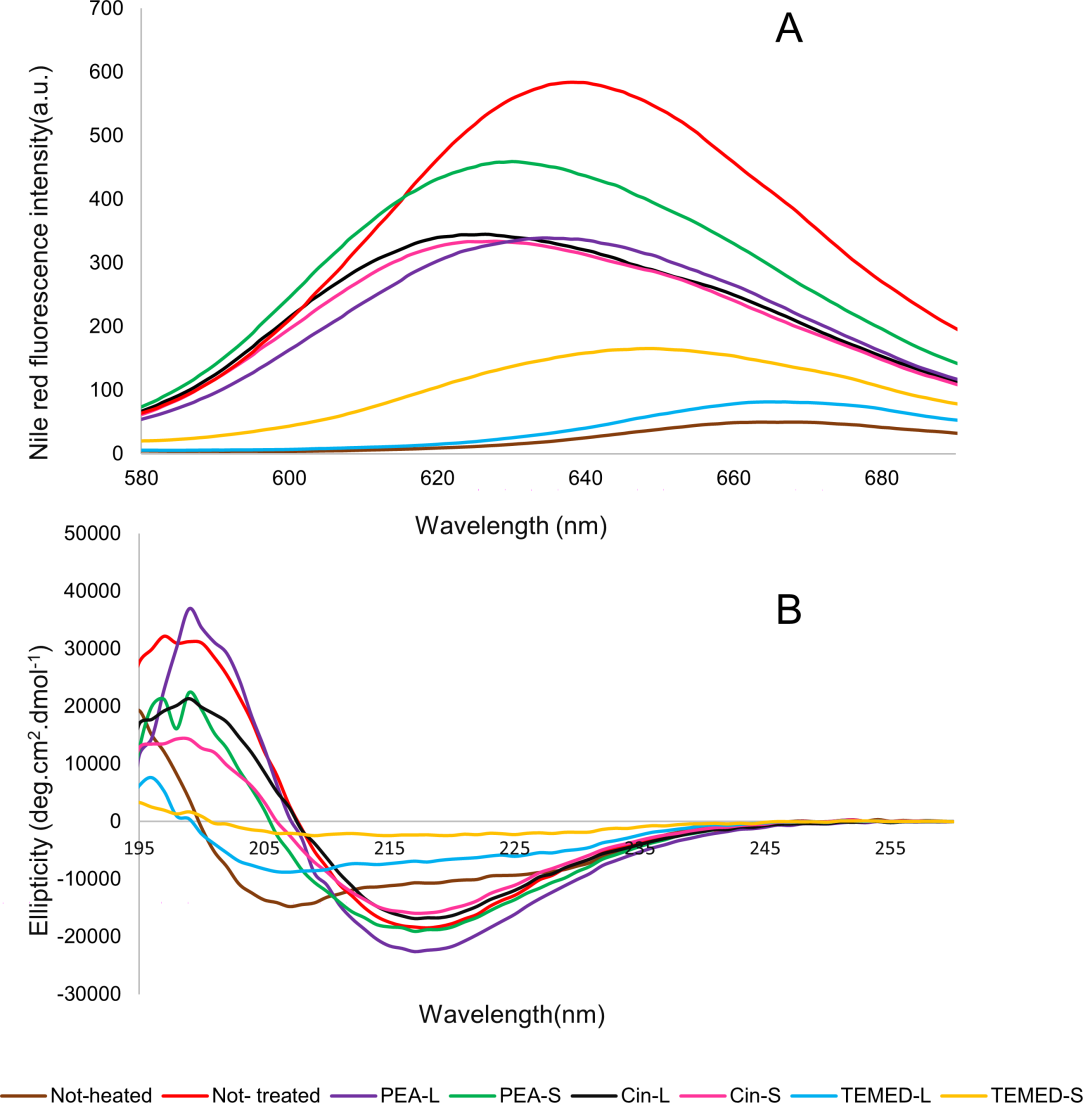
Analysis of hydrophobic patches and secondary structure of HEWL treated with aroma. (A) Effect of PEA, Cin and TEMED aroma on the surface hydrophobicity of HEWL. Changes in Nile red emission spectra obtained in the presence of HEWL at pH 2.2 when not-heated or incubated at 54°C with or without different aroma. Further details are given in the ‘Experimental procedures’ section of this manuscript. (B) Far-UV CD spectra of HEWL incubated with or without aroma. Secondary structures of not-heated HEWL in comparison with incubated HEWL after 24 hours with or without aroma.

To assess the effect of different aroma on the exposure of hydrophobic surfaces and secondary structure of HEWL, Nile red fluorescence spectroscopy and circular dichroism (CD) measurements were carried out. Nile red is an uncharged phenoxazone dye which interacts with exposed hydrophobic sites of proteins resulting in the enhancement of the fluorescence intensity and blue shifting of the emission maxima. Proteins which are known to have hydrophobic binding domains such as k-casein, 8-lactoglobulin and serum albumin exhibit greater enhancement and greater shifts of fluorescence than proteins which do not have such domains, such as lysozyme and ovalbumin. Therefore, Nile red can be used as a fluorescent probe of hydrophobic domains of proteins in their native state as well as monitoring conformational changes resulting in the formation or destruction of such domains, including partial denaturation of proteins, aggregation and unfolding of proteins [42]. Beta-sheet structures are composed of hydrophobic regions which can be detected by Nile Red. As the amount of beta-sheet content increases, the Nile Red fluorescence intensity too increases. Nile red fluorescence intensity measurements were taken of HEWL samples in glycine pH 2.2 incubated at 54°C, with or without aroma treatment (Fig 4A). The presence of PEA-S, PEA-L, Cin-S or Cin-L resulted in reduced exposure of hydrophobic patches compared to the not-treated HEWL sample with the highest exposure of hydrophobic patches when HEWL becomes fibrillar with beta sheet structures. However, neither PEA nor Cin were able to retain the native folded structure of HEWL as compared to the not-heated sample. Additionally, results showed that there was a small change in the Nile red fluorescence intensity for HEWL treated with TEMED-L compared to the not-heated HEWL control sample meaning that TEMED-L was able to maintain the native (not-heated) folded structure of HEWL monomers, such that the Nile red dye could no longer detect hydrophobic regions. However, TEMED-S was not able to fully hinder exposure of hydrophobic patches compared to the not-heated HEWL sample. Nevertheless, the exposure of hydrophobic patches in the presence of the aroma of the diamine, even in small amount (TEMED-S), was still reduced dramatically compared to the not-treated HEWL sample and those of HEWL treated with the aroma of PEA and Cin (minimal data set for Nile red fluorescence assay results is available in S2 Fig).

The secondary structure changes of HEWL in this study were assessed using CD measurements in the far-UV region of the spectrum (CD-UV). Fig 4B illustrates the CD spectra for not-treated and aroma-treated HEWL after incubation for 24 hours, under fibrillation conditions, in comparison with not-heated HEWL. The CD spectra of HEWL treated with the aroma of PEA and Cin, both despite their effectiveness in reducing the amount of hydrophobic patches detected, revealed no secondary structure changes compared to the not-treated HEWL and the same beta-sheet structure was seen, which was even more pronounced for HEWL treated with PEA-L aroma (Fig 4B). However, the secondary structure of HEWL in the presence of TEMED-L aroma was similar to the not-heated HEWL, which confirms that TEMED-L is able to maintain the secondary structure of HEWL and prevent amyloid fibril formation. Furthermore, the secondary structure of HEWL was changed into a partially unfolded structure when treated with TEMED-S. The CD results here indicate that TEMED-S may drive HEWL towards the unfolded form and hence the formation of amorphous aggregates (which may also explain the sample’s turbidity after 24 hours incubation compared to the other samples in this study; please see S3 Fig). It has been previously reported that amorphous aggregates of lysozyme cause turbidity in solution [43], which is similar to what we seen for the effect of TEMED-S aroma on HEWL upon incubation (minimal data set for circular dichroism spectroscopy results is available in S4 Fig).

### Analysis of changes in HEWL size using dynamic light scattering

**Fig 5:**
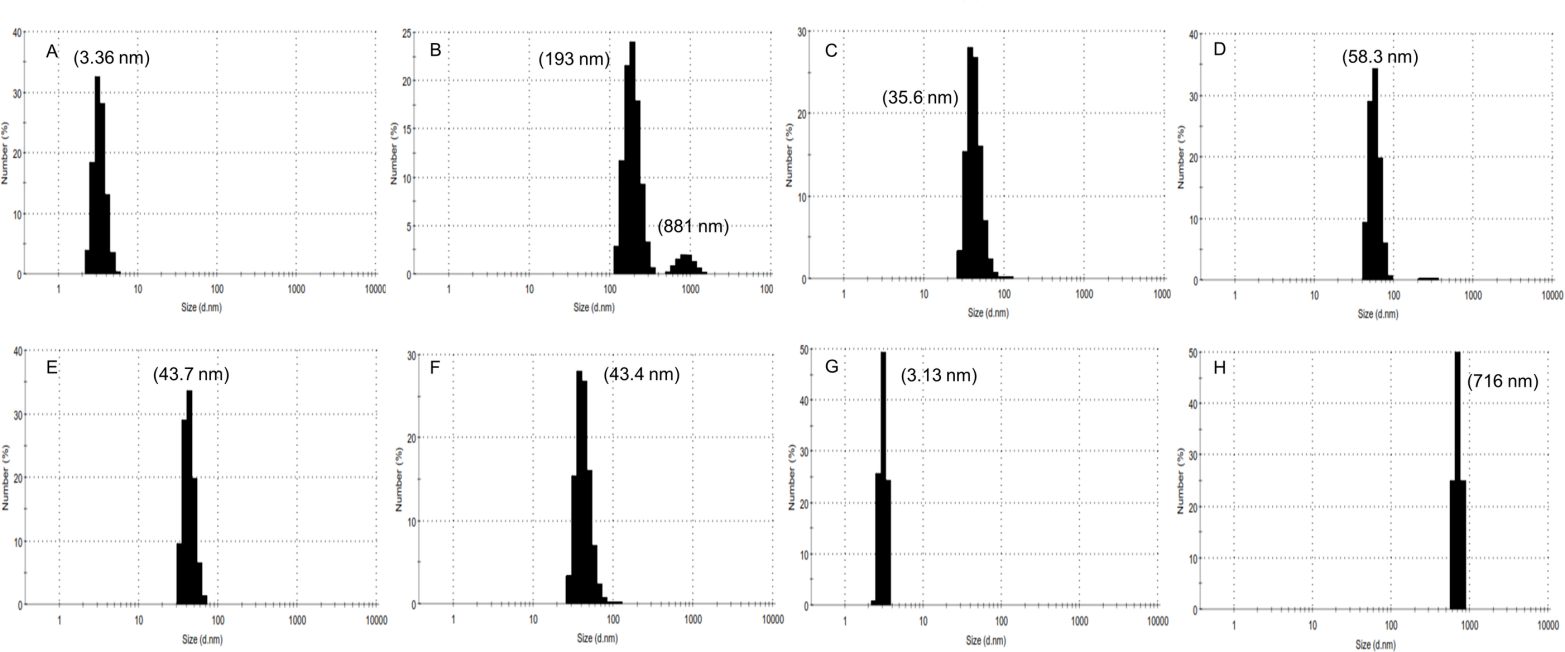
Size distribution of HEWL as revealed by DLS. The panels represent the DLS graphs of size distribution of HEWL particles for not-heated HEWL (A); not-treated HEWL incubated for 24 hours (B); HEWL treated with PEA-L aroma (C); HEWL treated with PEA-S aroma (D); HEWL treated with Cin-L aroma (E); HEWL treated with Cin-S aroma (F); HEWL treated with TEMED-L aroma (G); and HEWL treated with TEMED-S aroma (H). Measurements of HEWL sample solutions were done at 2 mg/ml.

Dynamic light scattering (DLS) results have shown that the diameter of not-heated HEWL was shifted from 3.36 nm to 193 nm in the heated HEWL in the absence of aroma (i.e. the not-treated sample) upon 24 hours of incubation under fibrillation conditions (Fig 5A and 5B). The effect of aroma of PEA and Cin in small and large amounts were shown to reduce the rate of fibril formation as clearly revealed by DLS results. Interestingly HEWL treated with PEA-L and PEA-S had the smallest and biggest diameter sizes of 35.6 nm and 58.3 nm, respectively. This trend is contrary to the results seen in ThT fluorescence assay where PEA-L had the least reduction in ThT fluorescence intensity compared to the positive control. This therefore suggests that oligomers with beta sheet structures are formed when HEWL is treated with PEA-L, in line with the ThT and in particular CD results. In the case of HEWL treated with Cin-S and Cin-L, both revealed a similar size in DLS with diameter sizes being 43.7 nm and 43.4 nm, respectively. These results are in agreement with ThT, Nile red and CD results. It is suggested that a mixture of protofibrils and mature fibrils are present in samples of HEWL treated with PEA-S, Cin-S and Cin-L (as shown in DLS data in the intensity mode; please see S5 Fig). In the case of TEMED-L, however, the diameter of HEWL was retained and even became more compact at 3.13 nm (Fig 5G) compared to the not-heated HEWL. DLS results, therefore, showed that TEMED-L was able to prevent fibril formation and supported the previous results seen using ThT, Nile red and CD analyses. On the other hand, as expected, TEMED-S resulted in a very large diameter of 716 nm as the HEWL is driven to an unfolded structure and the formation of amorphous aggregates, again in line with previous results (Fig 5H).

### Visualization of HEWL fibrils by atomic force microscopy (AFM)

**Fig 6:**
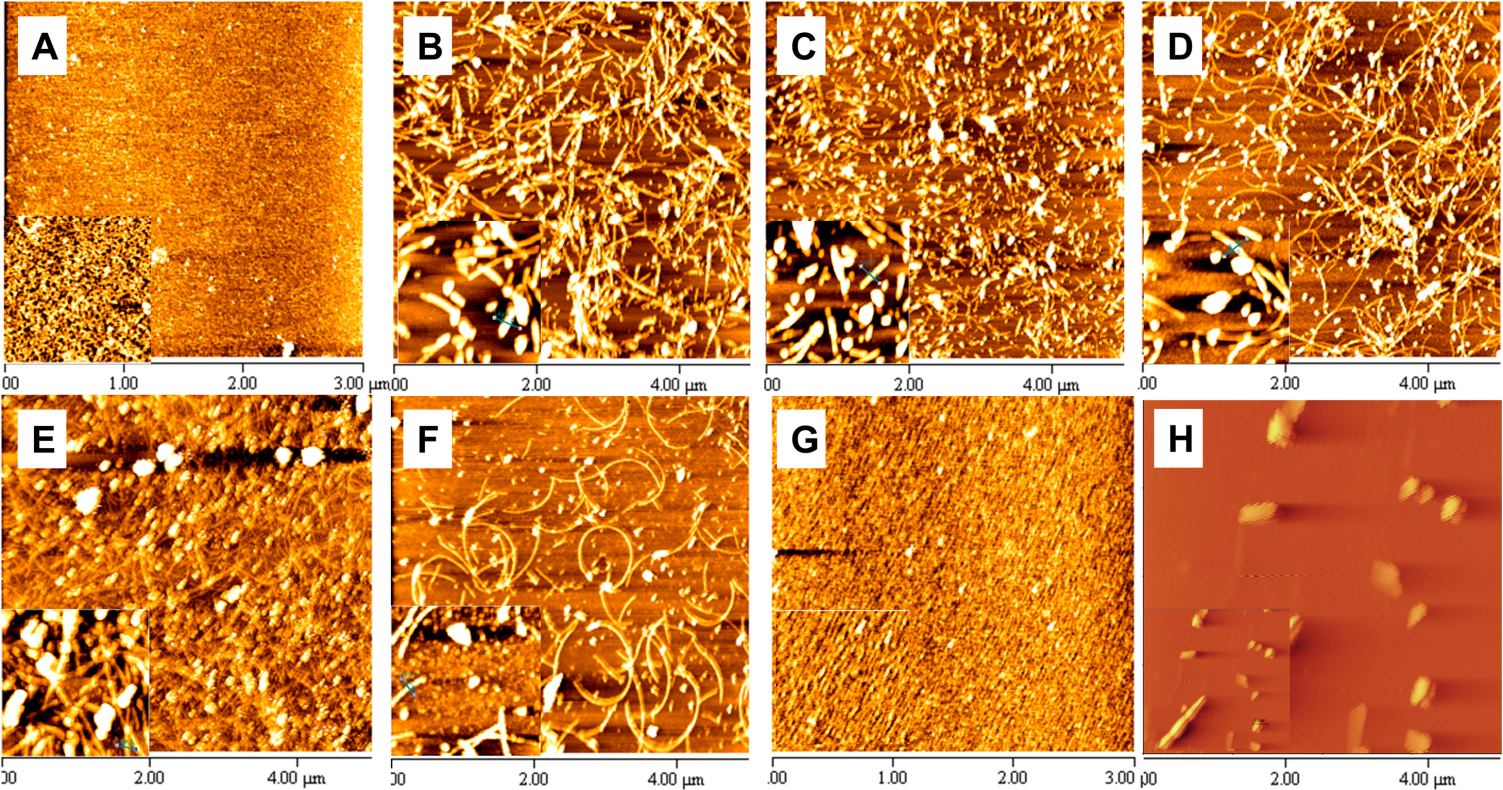
AFM analysis of HEWL incubated with or without aroma. AFM images of not-heated HEWL (A); not-treated HEWL (B); HEWL treated with PEA-L aroma (C); HEWL treated with PEA-S aroma (D); HEWL treated with Cin-L aroma (E); HEWL treated with Cin-S aroma (F); HEWL treated with TEMED-L aroma (G); and HEWL treated with TEMED-S aroma (H).

AFM images confirm previous results since in the absence of aroma (in the not-treated HEWL sample) fibrils with width size of 8.54 nm were seen (Fig 6B) but when HEWL was treated with PEA or Cin (Fig 6C, 6D, 6E and 6F), a clear reduction in fibril accumulation and their width size was detectable; fibril width sizes were reduced to 3.419, 2.518, 3.22 and 2.045 nm in HEWL treated with PEA-L, PEA-S, Cin-L and Cin-S, respectively. Interestingly, despite the width size reported, AFM images indicate the presence of non-fibrillar oligomers in HEWL treated with PEA-L in addition to short protofibrils, compared to a more protofibrillar form seen in HEWL treated with PEA-S. This is in line with Nile Red and DLS results which also reveal that PEA-L has done a better job of preventing fibril formation since PEA-L has a lower surface hydrophobicity and a smaller diameter size at 35.6 nm compared to PEA-S with higher surface hydrophobicity and a diameter size of 58.3 nm. The diameter size in HEWL treated with PEA-S is larger due to the presence of a mixture of protofibrils and mature fibrils. As for the effect of aroma from Cin-L and Cin-S, the results are similar and in line with previous data suggesting that they both direct HEWL mainly towards protofibril formation. In the case of HEWL treated with TEMED-L or TEMED-S, no fibrils were detected, also in agreement with previous results obtained. Results show that TEMED prevents HEWL from going down the path of fibril formation and instead either retains HEWL's native structure (TEMED-L) or directs it towards amorphous aggregate formation (TEMED-S), as supported by CD and DLS results, in particular.

### Enzyme activity assay

**Fig 7:**
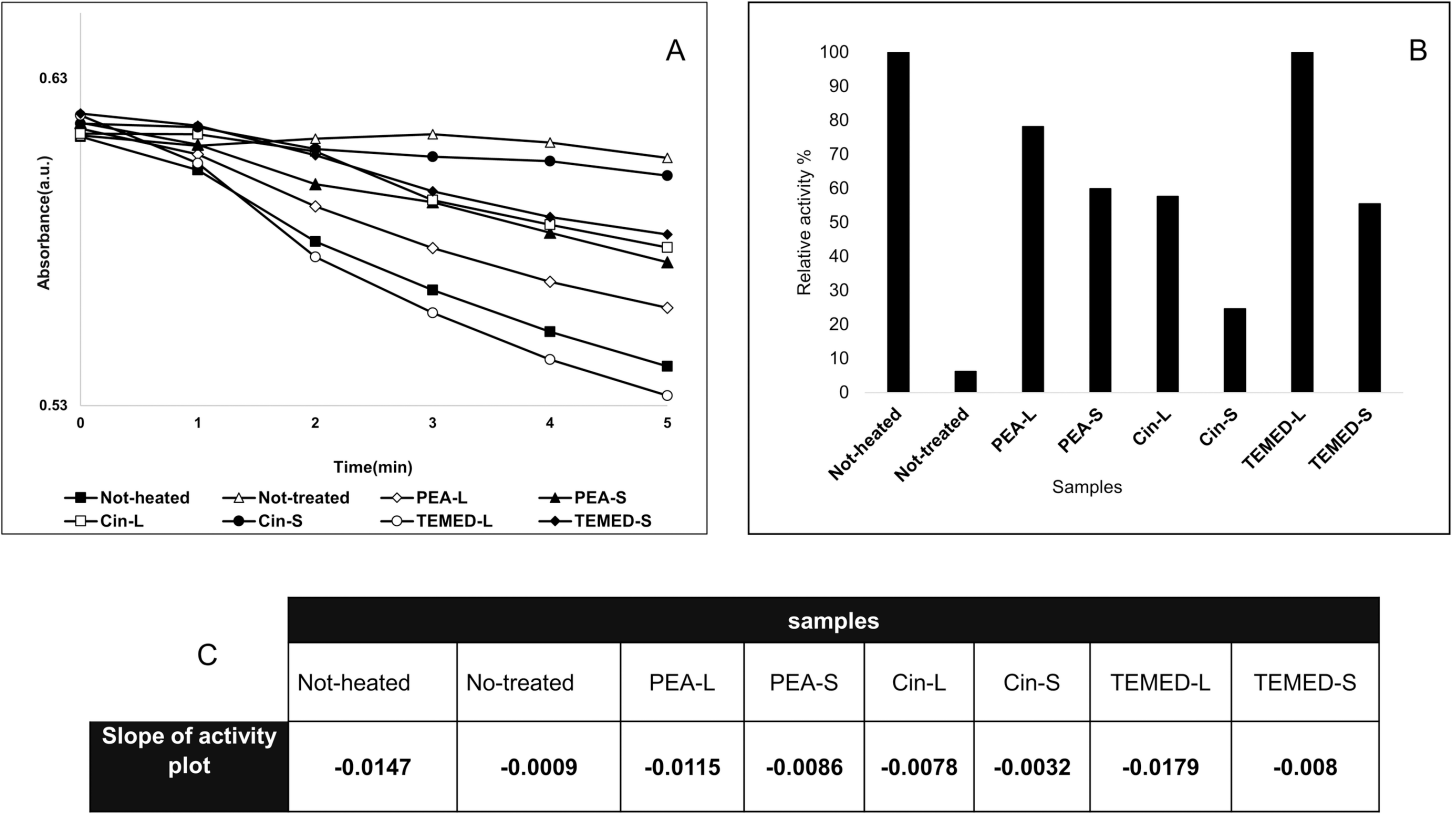
Enzyme activity assay. (A) Rate of lysis of *Micrococcus lysodeikticus* (*M*. *luteus*) as a function of HEWL, pH = 6.2, 25°C. (B) The relative activity (%) (With respect to the not-heated enzyme) of HEWL with or without varying aroma treatments. (C) Changes in the slope of activity plot as a representative of enzyme activity in different conditions.

To examine whether treatment with different aroma also retains the correct fold of the protein, enzymatic activity of HEWL was assessed and compared to the not-heated active HEWL form (Fig 7A). The assay was performed using *Micrococcus luetus* as HEWL's substrate. The slope of the activity plot is an indicator of the enzyme activity (Fig 7C). The results show that treatment with aroma retains HEWL activity compared to the not-treated HEWL. Interestingly HEWL treated with PEA-L aroma was able to retain more activity compared to the PEA-S, Cin-S and Cin-L (Fig 7B). This can be supported by our proposal of the presence of an oligomeric intermediate which still shows reasonable activity compared to protofibrils and mature fibrils. As for the effect of TEMED aroma on HEWL activity, TEMED-L surprisingly had more activity than not-heated HEWL. The activity of HEWL treated with the aroma of TEMED-S was similar to the not-treated sample (minimal data set for enzyme activity assay results is available in S6 Fig).

### Effect of aroma on HEWL as assessed by SDS-PAGE

**Fig 8:**
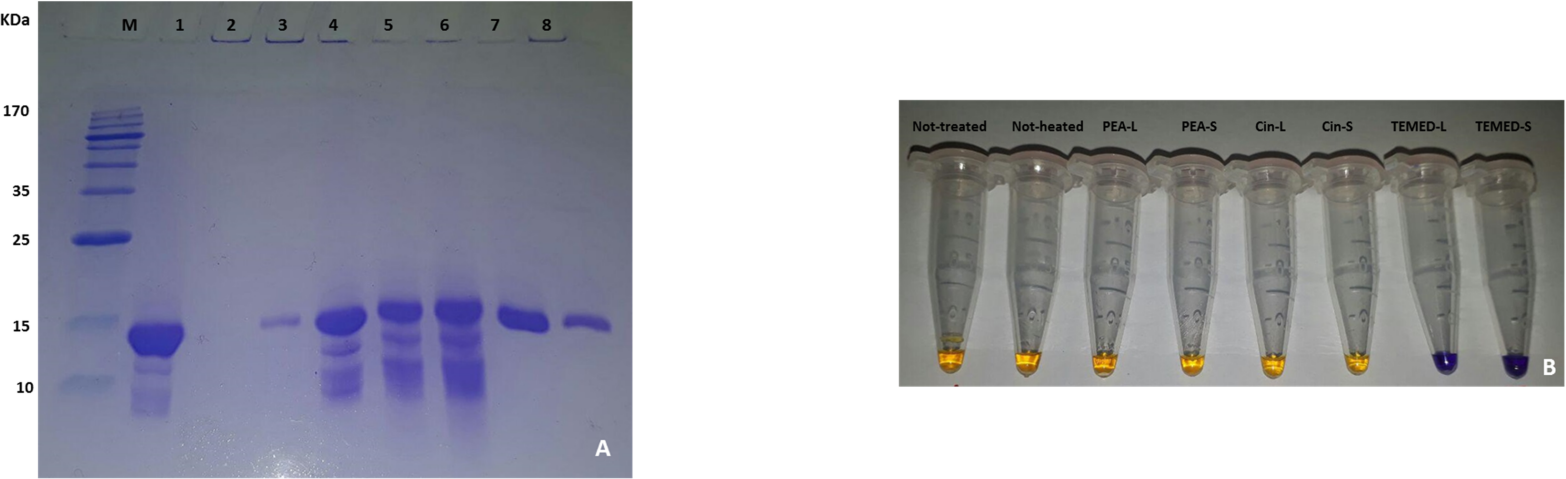
Effect of aroma of HEWL as assessed by SDS-PAGE. (A) Gel lanes are as follows: Protein marker (M), not-heated HEWL (1), not-treated HEWL (2), HEWL treated with different aroma (lanes 3–8); PEA-L, PEA-S, Cin-L, Cin-S, TEMED-L and TEMED-S, respectively). (B) Colour of samples 1–8 with added loading dye for SDS-PAGE analysis.

The next step was to determine the effect of aroma on HEWL through SDS-PAGE analysis (Fig 8B). It was observed that not-heated HEWL migrated well into the gel just below the 15 kDa protein marker (HEWL has a molecular weight of 14.3 kDa). The not-treated HEWL however did not enter the gel fully and only a thin band was seen. This can be explained by the fact that mature fibrils are formed and an 18% gel is used, hindering migration into the gel. As for HEWL treated with aroma, relatively strong bands compared to the not-treated sample were seen, meaning that the aroma interaction was able to inhibit mature fibril formation and allow entry of protofibrils into the SDS polyacrylamide gel. The aroma however was seen to have varying effects as seen in previous data. The HEWL band seen when treated with PEA-L was less intense than HEWL bands seen when treated with PEA-S, Cin-L and Cin-S, indicating the formation of a mixture of oligomers and protofibrils. It can thus be proposed that the portion of HEWL entering the gel represent the protofibrillar form and the portion unable to enter represent the oligomeric form. Therefore, it can be stated that while the native HEWL is able to enter the 18% gel, the HEWL forming insoluble mature fibrils cannot enter the gel and what seems to enter may be protofibrils with intermolecular interactions breakable under reducing SDS PAGE conditions. As for HEWL treated with PEA-L, a mixture of protofibrils and oligomers are formed, with the protofibrils capable of entering the gel, but the oligomers being too large to migrate into the gel (hence the reduced intensity of HEWL band in lane 3, Fig 8B). In HEWL treated with PEA-S, a mixture of protofibrils and fibrils are formed, with the protofibrils entering the gel. HEWL treated with Cin-L and Cin-S show similar results where a mixture of protofibrils and fibrils are detected. As for TEMED, HEWL treated with TEMED-L is able to almost fully enter the gel, being folded and retaining the native form of HEWL, however, a smaller portion of HEWL treated with TEMED-S is seen to enter the gel since we propose the existence of a mixture of misfolded structures and amorphous aggregates of HEWL to be present. The amorphous aggregate form is not able to enter the gel leaving the misfolded structures of HEWL to enter the gel (Fig 8B). Additionally, as an interesting observation is the presence of a number of low molecular weight (LMW) bands for HEWL in lanes 1–6 and not 7 and 8. Most amazingly, the 'aroma' of TEMED was able to change the pH environment of HEWL from acidic (pH 2.2) to basic (about pH 8.0) and hindered degradation of HEWL (as no LMW bands are seen in these samples). This was also evident in the colour of the samples prepared for running the SDS-PAGE where the blue colour of the loading dye was retained in the HEWL samples treated with TEMED aroma but not in the not-treated or PEA and Cin aroma treated HEWL samples (Fig 8B) (which all turned orange instead due to their acidic pH of about 2.2).

## Discussion

In this study the novel effects of aroma from three different compounds including a phenol, an aldehyde and a diamine in inhibiting HEWL fibril formation has been revealed. The compounds have different inhibitory effects on the fibrillation pathway such that the phenol and aldehyde were able to partially prevent fibril formation and produce mainly oligomers (PEA-L) and protofibrils (PEA-S, Cin-S and Cin-L), while the diamine was capable of fully retaining HEWL's native structure (TEMED-L) or directing HEWL towards misfolded or amorphous aggregate formation (TEMED-S). Several different techniques had to be used to derive at these conclusions.

The results seen for the aroma of the phenol and aldehyde with a single aromatic ring using different techniques were as follows: ThT results revealed that the aroma of all the compounds studied here were able to reduce mature fibril formation compared to the not-treated samples, however, the major discrepancy seen in PEA-L can be explained by the study of Hudson et al (2009) on the effect of presence of exogenous compounds on ThT fluorescence assay. They state that phenolic aromatic rings of polyphenols are often connected via conjugated systems, resulting in strong ח-ח* electronic transitions and making them chromophoric and even intrinsically fluorescent at the same wavelength of ThT fluorescence [44]. Hence PEA-L, being a phenol in large amounts, in particular, may have shown this effect making ThT assay unsuitable for this sample specifically. Therefore, other techniques were used to assess the effect of PEA-L aroma.

Hydrophobic patch analysis results show that the Nile red fluorescence intensity increases as the hydrophobic patches are exposed on the surface of HEWL. The not-treated HEWL had the most intense Nile red fluorescence compared to the not-heated sample since the beta sheet structure is more hydrophobic than the native structure. The aroma of PEA and Cin were able to reduce the exposure of hydrophobic patches compared to the not-treated sample, indicating their ability to prevent mature fibril formation.

CD spectra showed that the secondary structure of HEWL in the presence of the aroma of these aromatic compounds consists of beta-sheet structure (same as 'not-treated' HEWL). Additionally, CD results revealed a surprisingly higher beta-sheet structure content in HEWL treated with PEA-L (consisting of oligomers) in comparison to the 'not-treated' HEWL sample (consisting of mature amyloid fibrils). This can be explained by the fact that precursors of soluble aggregates are highly structured whereas amyloid fibrils have minimum structured conformations [5,45].

AFM and DLS results confirmed the formation of a mixture of oligomeric and/or protofibrillar and mature fibril structures when HEWL was treated with aroma of these aromatic compounds. This was in line with other results seen. As for SDS-PAGE analysis of the samples, the analysis was based on earlier studies showing that protofibrils often have curvilinear shape and are shorter and narrower than mature fibrils [46] and hence capable of entering the SDS polyacrylamide gel. Therefore, in this study, the protofibrillar form of HEWL in addition to the native form and misfolded structure were able to enter the gel leaving the amorphous aggregates, oligomers and mature fibrils behind. The reduced level of entry of HEWL treated with PEA-L into the SDS-PAGE, compared to PEA-S, Cin-L and Cin-S, also confirms the presence of the oligomeric form as the oligomer is considered too large to enter compared to the narrow curvilinear shaped protofibrils. As for activity test, HEWL treated with PEA-L had more activity than HEWL treated with PEA-S, Cin-L and Cin-S and again can be explained by the existence of the oligomeric form of HEWL compared to the protofibrillar and fibrillar HEWL forms.

As for the diamine, all the techniques used here supported the finding that TEMED-L was able to retain the native form of HEWL and completely prevented fibril formation. TEMED-S aroma, however, directed HEWL away from the fibril formation pathway and formed amorphous aggregates, again supported by the techniques used in this study. As it has been shown in previous studies, multiple amine groups and short alkyl chains in diamines play critical roles in preventing thermal aggregation and inactivation of lysozyme [24]. It was also hypothesised that the difference in the effect of diamines in the prevention of thermal aggregation and inactivation is due to a hydrophobic effect rather than a simple electrostatic consideration. It seems plausible that in our study too, TEMED (as a diamine), with no positive charges, may cause inhibition of HEWL fibril formation by the hydrophobic effect of the alkyl chain. This can be explained by the reasons described by Kudou et al (2005) [23] stating that if the effective concentration of the positive charge in a solution plays an essential role in the prevention of thermal aggregation and inactivation, different concentrations of polyamines should have almost identical results. However, this was not observed in their study. In line with the reasoning stated, our data show that, the two different concentrations of TEMED aroma (small and large amounts) did not show the same effects in preventing fibril formation.

Moreover, we propose that even the aroma of TEMED (with TEMED in the liquid form having a pH of 10.36) can decrease the acidity of HEWL solution environment by interacting with H^+^ ions and inhibiting degradation of HEWL as seen in SDS-PAGE for HEWL treated with TEMED (where no LMW bands are seen, Fig 8B) and also capable of maintaining HEWL's native structure (with activity test results showing comparable level of activity of HEWL treated with TEMED-L with that of not-heated HEWL, Fig 7). This is also supported by preservation of the blue colour of the SDS-loading dye in the HEWL samples treated with TEMED as compared to the rest of the HEWL samples (including not-heated, not-treated, and treated with polyphenols) where the loading dye colour was changed to orange because of the acidic HEWL solution environment of glycine at pH 2.2 (Fig 8A). The pH of all HEWL samples post-incubation was checked showing that the 'not-treated' and PEA and Cin treated samples were still at the starting acidic pH of 2.2, while TEMED-treated HEWL showed an amazing dramatic pH change from 2.2 to about 8.0. It can be said that the nitrogen atoms in TEMED act as hydrogen acceptors similar to the nitrogen ions of a well-known osmolyte (TMAO) used for investigating HEWL’s stability, which interact with H^+^ ions in acidic environments (raising the pH) and shortening the water H-bonds leading to the promotion of tighter protein folding (better thermal stability) [47]. The latter property is also seen in TEMED and supported by DLS results showing that TEMED-L is able to make HEWL more compact with a diameter of 3.13 nm compared to the not-heated HEWL (native HEWL) with a diameter of 3.36 nm (Fig 5).

## Conclusions

This research has unraveled novel data showing the effects of aroma from three small molecules in inhibiting HEWL fibril formation. This is the first time, the effect of aroma has been studied at the molecular level in preventing amyloid fibril formation using HEWL as a model protein in studying protein stability, folding and aggregation.

**Fig 9:**
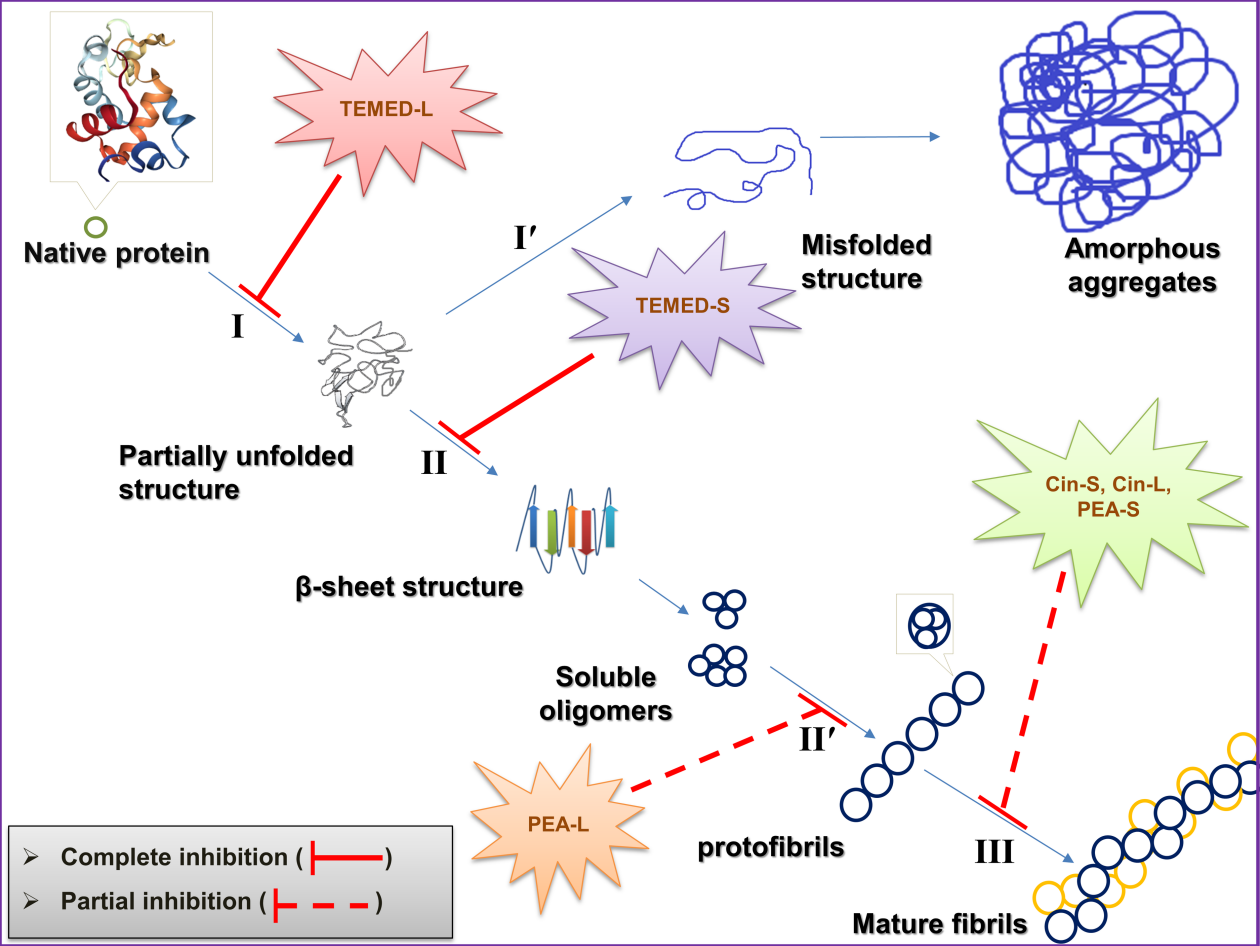
Graphical abstract. A possible mechanism of the effect of aroma from a phenol, an aldehyde and a diamine in preventing HEWL fibril formation. All pathways were concluded using results from several different experiments.

The data from this study suggest different pathways for preventing or inhibiting amyloid fibril formation by the aroma of three different small molecules used in this study (Fig 9). It can be said that PEA being a major constituent of *Rosa damascene* in producing odour has been recognised to prevent mature fibril formation since the PEA-L aroma is able to trap the oligomeric form of HEWL in contrast to PEA-S aroma where protofibrils but not mature fibrils were formed. The possibility to trap oligomeric species along the fibrillation pathway by PEA-L aroma is of importance for further research as it provides routes for preventing the formation of toxic oligomeric and or other unwanted intermediates along the fibrillation pathway. Additionally, previous research have suggested the presence of at least two phenolic rings each with three hydroxyl groups as essential in preventing fibril formation but results here show that PEA with just one phenolic ring and a single hydroxyl group may be sufficient even in the aroma form.

As for cinnamaldehyde, it was also recognised to prevent fibrillation in the aroma form where predominantly protofibrils and not mature fibrils were formed in both small and large amounts. It is plausible to say that aromatic groups of PEA and Cin may be involved in stacking between beta-sheet structures to prevent fibril formation [1,13,18,19]. However, the exact mechanisms at work are to be explored.

TEMED on the other hand, was able to fully retain the native form in TEMED-L and completely stop the fibrillation process, while TEMED-S took the route of amorphous aggregate formation. The nitrogen atoms in TEMED are important and may act as hydrogen acceptors interacting with H^+^ ions in acidic environments and shortening the water H-bonds leading to the promotion of tighter HEWL folding (seen in TEMED-L) similar to TMAO [47]. Furthermore, it seems that TEMED may cause inhibition of HEWL fibril formation by the hydrophobic effect of the alkyl chain and interact directly with HEWL by its alkyl chain. Again, the exact mode of interaction and mechanism is to be derived by future structural studies.

In conclusion, considering that amyloid fibrils are toxic, any inhibitor which can hamper amyloid fibril formation could potentially be considered as a way for delaying and/or inhibiting amyloid formation. Additionally, identification of these small aroma producing molecules and the results showing their effect in inhibiting the fibril formation pathway could help improve our knowledge about the very important neurodegenerative disease-related aggregation, misfolding and fibrillation pathways.

## Supporting information

S1 Fig Minimal data set of ThT fluorescence assay. (XLSX)

S2 Fig Minimal data set of Nile red fluorescence assay. (XLSX)

S3 Fig Formation of amorphous aggregates revealed by difference in samples appearance. Turbidity seen in HEWL treated with TEMED-S after 24 hours incubation compared to the other samples.
(TIF)

S4 Fig Minimal data set of Circular Dichroism Spectroscopy. (XLSX)

S5 Fig Size distribution of HEWL as revealed by DLS in intensity mode. A mixture of protofibrils and mature fibrils are present in samples of HEWL treated with PEA-S (a), Cin-S (b) and Cin-L (c).
(TIF)

S6 Fig Minimal data set of enzyme activity assay. (XLSX)
