# Supplementary figures and images for: Unraveling the novel effects of aroma from small molecules in preventing hen egg white lysozyme amyloid fibril formation
Source: PLoS One. 2018 Jan 22;13(1):e0189754. doi: 10.1371/journal.pone.0189754 (PMC5777642; doi:10.1371/journal.pone.0189754)

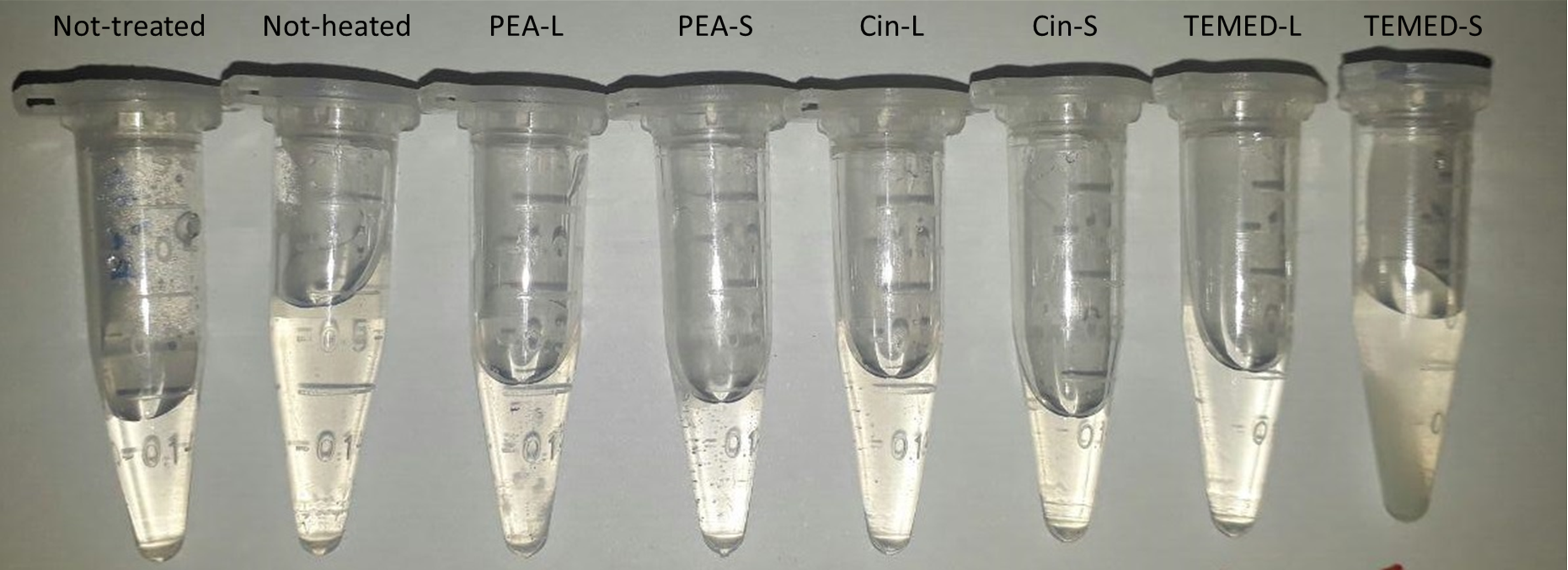

Supplement: S3 Fig — Turbidity seen in HEWL treated with TEMED-S after 24 hours incubation compared to the other samples. (TIF) [file pone.0189754.s003.tif]

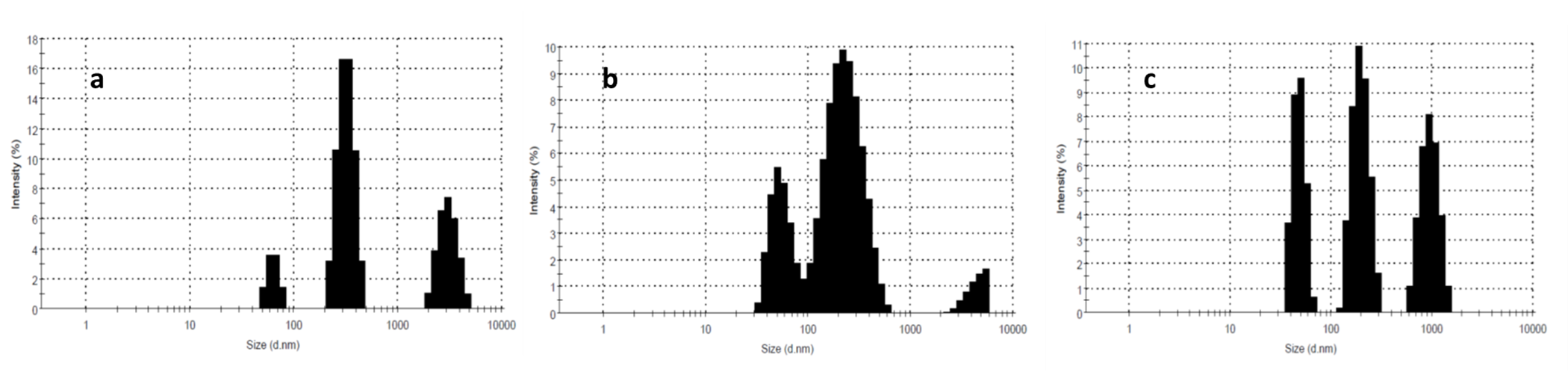

Supplement: S5 Fig — A mixture of protofibrils and mature fibrils are present in samples of HEWL treated with PEA-S (a), Cin-S (b) and Cin-L (c). (TIF) [file pone.0189754.s005.tif]
